# Effects of Aging on Z-DNA-Induced Genetic Instability In Vivo

**DOI:** 10.3390/genes16080942

**Published:** 2025-08-11

**Authors:** Tonia T. Li, Alexandra D’Amico, Laura Christensen, Karen M. Vasquez

**Affiliations:** Division of Pharmacology and Toxicology, Dell Pediatric Research Institute, College of Pharmacy, The University of Texas at Austin, 1400 Barbara Jordan Boulevard, Austin, TX 78723, USA; tonia.li@utexas.edu (T.T.L.); amdamico@utexas.edu (A.D.); laura.christensen@austin.utexas.edu (L.C.)

**Keywords:** DNA repair, non-B DNA, cancer, genetic instability, aging

## Abstract

Repetitive DNA sequences are abundant in genomes and can adopt alternative DNA structures (i.e., non-B DNA). One such structure, Z-DNA, has been shown to stimulate genetic instability in a variety of organisms, including human cells and mice. Z-DNA-forming sequences are enriched at mutation hotspots in human cancer genomes, implicating them in cancer etiology. Aging is a known risk factor for the development of cancer, and genetic instability is a hallmark of both aging and cancer. However, how aging affects the mutagenic potential of Z-DNA has not yet been investigated. Here, we explored the effects of aging on the mutagenic processing of Z-DNA using a transgenic mouse model. Surprisingly, Z-DNA-induced mutations decreased or remained unchanged with increasing age. Cleavage of Z-DNA was unaffected with increasing age, suggesting that downstream repair processing, such as double-strand break repair processes, may be involved in the age-related changes in Z-DNA-induced mutagenesis in mice.

## 1. Introduction

A wide variety of diseases are associated with aging, including, but not limited to, heart disease, neurological disorders, osteoporosis, and cancer. Currently, there is a global increase in the population of aging individuals due to longer life expectancies and decreases in fertility [1,2]. With an aging population, the probability of developing diseases increases [3,4,5]. Thus, it is critical to gain a better understanding of the mechanisms involved in aging-related disease development.

Cancer remains a disease associated with aging, as the cancer incidence rate increases from 1.0% at ages less than 20 to 28.2% at ages 65–74 at all sites of cancer development [3]. Genetic instability is a leading hallmark in cancer development [6,7,8] and can be caused by both endogenous [9,10,11,12] and exogenous sources of DNA damage [12,13]. Alternative DNA structures (e.g., Z-DNA) are an example of an endogenous source of genetic instability that has been linked to the development of cancer [9].

Z-DNA is a left-handed DNA duplex that forms at alternating purine and pyrimidine repeats [14,15] and is stabilized by negative supercoiling that is generated during cellular replication and transcription [16,17,18,19]. Z-DNA-forming sequences are estimated to occur at approximately 1 in every 3000 bp in the human genome and are significantly enriched at mutation hotspots in human cancer genomes [20,21,22,23,24]. Z-DNA has been shown to be mutagenic in yeast, bacteria, mammalian cells, and mice [25,26,27]. Notably, Z-DNA has been implicated in translocation breakpoints in human cancer genomes [23] and has been found in the hippocampus of brains affected with Alzheimer’s disease [28,29].

Recently, we identified a mechanism for the processing of Z-DNA using human cells in which the DNA repair protein complexes from the mismatch repair and nucleotide excision repair pathways, MSH2-MSH3 and ERCC1-XPF, respectively, recognize and cleave Z-DNA structures, resulting in DNA double-strand breaks (DSBs) [25]. Although it is known that Z-DNA is mutagenic [25,26,27] and is implicated in the development of disease(s) [20,21,22,23,24,28,30], how aging affects the mutagenic potential of Z-DNA has not been determined. Here, we explored how aging impacts the mutagenic processing of Z-DNA in vivo, using male transgenic mice containing either Z-DNA or canonical B-DNA-forming sequences within a recoverable mutation reporter [27]. Interestingly, we found either no changes or decreases in Z-DNA-induced mutations with increasing age in our target tissues (i.e., brain, liver, spleen, and testis). Further analysis suggests that alterations in DSB repair pathways with age are responsible for the observed decreases in Z-DNA-induced mutation frequencies with increasing age.

## 2. Methods

**Mice.** Transgenic mice used in this experiment were established as previously described [27]. Briefly, male FVB/N mice containing a chromosomally integrated lacZ mutation reporter with either a B-DNA sequence or a Z-DNA-forming sequence (CG14-repeat) were used. The mice were aged to 2 months and 18 months. Three to six mice were used per group for statistical analysis, with each mouse serving as a biological replicate. The mice were housed and handled according to IACUC guidelines.

**LacI-LacZ Magnetic Beads for Mutation Reporter Recovery.** LacI-LacZ magnetic beads were prepared as previously described [31]. Briefly, Dynabeads Sheep anti-Mouse IgG (Invitrogen, Carlsbad, CA, USA, REF: 11031) was conjugated to anti-β-galactosidase monoclonal antibody (Promega, Madison, WI, USA, REF: Z3783). The magnetic beads were then washed with 1× PBS before being resuspended in 960 μL PBS. Forty μL lacI-lacZ fusion protein (~40 mg/mL) was added to the bead suspension, and the mixture was rotated at 37 °C for 2 h. After, the magnetic beads were washed and resuspended in 1× PBS and stored at 4 °C.

**Z-DNA-Induced Mutation Frequency Assays.** Male transgenic mice containing a recoverable B-DNA or Z-DNA mutation reporter [p2RT-CON (B-DNA) and p2RT-CG14 (Z-DNA)] were aged to 2 months and 18 months [27]. Upon reaching the target age, the brain, liver, spleen, and testis tissues were collected, and the mutation reporter was isolated as previously described [32]. Briefly, genomic DNA was isolated from the mouse tissues and digested with SpeI-HF enzyme (New England Biolabs, Ipswich, MA, USA, REF: R3133L). The genomic DNA was then purified via phenol–chloroform extraction and ethanol precipitation before being resuspended in 60 μL 1X binding buffer (5× binding buffer: 50 mM Tris-HCl, 5 mM EDTA, 50 mM MgCl_2_, 25% glycerol, pH = 6.8). To isolate the mutation reporter from genomic DNA, the DNA suspension was used to resuspend 70 μL lacI-lacZ magnetic beads and rotated at 37 °C for 1 h. Next, the magnetic beads were washed with 1× binding buffer before they were resuspended in elution buffer (75 μL IPTG EB solution: 10 mM Tris-HCl pH = 7.6, 1 mM EDTA, 125 mM NaCl, 5 μL 20 mg/mL IPTG, 20 μL NEBuffer2, and 100 μL nucleotide-free diH_2_0). To elute the mutation reporter from the magnetic beads, the mixture was rotated for 30 min at 37 °C and then for 20 min at 65 °C. Subsequently, the samples were treated with 40 U NEB T4 ligase (400 U/μL) and 1 μL 10 mM ATP for 1 h at 25 °C to ligate the mutation reporter into a plasmid. The magnetic beads were then pelleted and the supernatant containing the eluted mutation reporter was transferred a new tube. The eluted mutation reporter was then precipitated via ethanol precipitation and later resuspended in 10 μL of diH_2_O. The isolated mutation reporter was then treated with 2× Blunt/TA ligase master mix (NEB, Ipswich, MA, USA, REF: M0367L) for one hour at 25 °C, purified via phenol–chloroform extraction and ethanol precipitation, and resuspended in 10 μL of 10 mM Tris-HCl pH = 8.5 and stored at 4 °C.

**Isolation of Primary Mouse Fibroblasts.** Skin from the underarm area of 2-month-old and 18-month-old male FVB/NCr mice was collected and placed in PBS on ice. The skin tissue was cut further into small sections and air dried for 10–15 min in tissue culture dishes to increase adhesion. Subsequently, culture medium (DMEM/F12, 15%FBS, amphotericin, and penicillin–streptomycin) was added and the skin tissues were incubated at 37 °C in a low O_2_ incubator (5% O_2_). Once the cells reached 50–70% confluency, they were passaged using EMEM media containing 15% FBS, penicillin–streptomycin, and glutamine.

**Transfection of Z-DNA Mutation Reporters into Primary Mouse Fibroblasts.** The mutation reporters pYZ-Con (B-DNA) or pYZ-CG14 (Z-DNA) were transfected into primary mouse fibroblasts using a 4D-Nucleofector device (Lonza, Walkersville, MD, USA) and a P2 Primary Cell 4D-Nucleofector X Kit L using program CA-158 according to the manufacturer’s instructions. The cells were incubated for 72 h to allow for replication at 37 °C with 5% O_2_, and then the mutation reporters were collected and treated with DpnI to remove any reporters that had not replicated in the mouse cells.

**Blue–White Screening Analysis.** Recovered p2RT-CON/CG14 mutation reporter plasmids were transformed into ElectroMAX DH10β cells (Invitrogen, Carlsbad, CA, USA, REF: 18290015) and pYZ-Con/CG14 mutation reporters were transformed into MBM7070 cells to screen for Z-DNA-induced mutations. The transformants were resuspended in 1 mL of SOC medium and incubated at 37 °C, 225 rpm, for one hour. After, the transformants were spread on LB agar plates containing 100 μg/mL of carbenicillin, 200 μg/mL X-gal, and 400 μg/mL of IPTG and incubated at 37 °C overnight. A mutation frequency was obtained by dividing the number of white (mutant) colonies by the total number of colonies (blue plus white) counted (>10,000 colonies per group).

**Mutation Spectra Analysis.** Mutant white bacteria colonies obtained from the blue–white screening assay were streaked onto LB agar plates containing 100 μg/mL of carbenicillin, 200 μg/mL X-gal, and 400 μg/mL of IPTG and incubated at 37 °C overnight. Five mL bacteria cultures were generated by inoculating single white colonies into LB containing 100 μg/mL carbenicillin and were incubated at 37 °C overnight. Mutation reporters were then recovered using the QIAprep spin miniprep kit (Qiagen, Germantown, MD, USA, REF: 27106) and submitted for Sanger sequencing. The p2RT-CON/CG14 mutation reporters were sequenced using the 548 primer or 201 primer (Supplementary Table S1). The pYZ-Con/pYZ-CG14 and polyT-Con/polyT-CG14 reporters were sequenced using the seqprim 189 primer (Supplementary Table S1).

**Tissue Extract Preparation.** Tissues were pulverized in liquid nitrogen using a mortar and pestle. Pulverized tissues were incubated in NP-40 buffer (150 mM sodium chloride, 1.0% NP-40, 50 mM Tris pH = 8.0) with protease and phosphatase inhibitors for 1–2 h at 4 °C. Prior to NP-40 buffer incubation, spleen tissues were pretreated with red blood cell lysing buffer (Sigma, St. Louis, MO, USA, REF: R7757-100ML). Tissue extracts were then centrifuged, and the supernatant of the extracts was aliquoted, snap frozen, and stored at −80 °C. The Pierce^TM^ BCA Protein Assay Kit (Thermo Scientific^TM^, Waltham, MA, USA, REF: 23225) was used to quantify protein concentration.

**Primer Extension.** A primer extension assay was performed as previously described to visualize cleaved Z-DNA [25]. Briefly, 200 ng PNU-CON or PNU-CG14 was incubated in reaction buffer (5 mM MgCl_2_, 40 mM HEPES-KOH pH = 7.8, 0.5 mM DTT, 2 mM ATP, 22 mM phosphocreatine, 0.34 mg/mL BSA, 50 ng/μL CPK) and 100 μg of tissue extract in a reaction volume of 50 μL for 30 min at 30 °C. After incubation, samples were treated with 2 μL 0.5 M EDTA and 2 μL 2 mg/mL RNAse A for 10 min at 37 °C followed by the addition of 2.5 μL 10 % SDS and 2.5 μL 20 mg/mL proteinase K at 30 min at 50–65 °C. DNA was then purified by phenol–chloroform extraction and ethanol precipitation and resuspended in nuclease-free diH_2_O for PCR extension using either the JMleft or JMright primers (Supplementary Table S1). The PCR products were then visualized on an agarose gel using SYBR™ Gold Nucleic Acid Gel Stain (Invitrogen, Carlsbad, CA, USA, REF: S11494). ImageJ 1.53t was used to quantify cleavage products.

**DNA End-Joining Assay.** DNA substrates were constructed using restriction enzymes. Briefly, DNA products with overhanging ends were constructed by linearizing pNUCON plasmid DNA with EcoRI-HF (NEB, REF: R3101). To construct a DNA substrate with both an overhanging end and a blunt end, pNUCON plasmid DNA was dual digested with EcoRI-HF and SfoI (NEB, REF: R0606). For DNA substrates that were dual digested, the plasmids were run on a 1% agarose gel, and the large fragment (1786 bp) was gel purified using the Qiaquick Gel Extraction Kit (Qiagen, REF: 28704). In total, 100 ng of DNA substrates were incubated in reaction buffer (final concentration: 45 mM HEPES-KOH pH = 8.0, 7.5 mM MgCl_2_, 1 mM DTT, 2.5 mM ATP, 200 μM dNTPs, 10 μg/mL BSA, 2% glycerol, 22 mM phosphocreatine, and 50 ng/μL CPK) and 100 μg of tissue extracts for 4 h at 30 °C in a 50 μL reaction volume. After incubation, samples were treated with 2 μL 0.5 M EDTA and 2 μL 2 mg/mL RNAse A for 10 min at 37 °C followed by 2.5 μL 10 % SDS and 2.5 μL 20 mg/mL proteinase K at 30 min at 50–65 °C. The DNA was then purified by phenol–chloroform extraction and ethanol precipitation, resuspended in nuclease-free diH_2_O, and visualized on an agarose gel. Image J was used to quantify end-joined products.

**Immunohistochemistry.** Testis tissue from young (2-month-old) and aged (18-month-old) mice were harvested and fixed in 10% formalin. Paraffin block processing and slide preparation (3–5 µM) was performed by the STRL Histology/Immunohistochemistry Laboratory at the University of Texas Health San Antonio. The slides containing the testis tissue sections were prepped for antigen retrieval. Next, tissue sections were incubated in 3% hydrogen peroxide for 10 min and then washed twice with diH_2_O for 5 min each. A 2.5% Normal Horse Serum (Vector Laboratories, Newark, CA, USA, REF: MP-7500) was used to block tissue sections for 1 h at room temperature before the primary antibody (1% BSA in 1× PBST) was used. Tissue sections were then incubated in the primary antibody for 1 h at room temperature. ImmPRESS Universal Antibody (anti-mouse IgG/anti-rabbit IgG, Peroxidase) Polymer Reagent (Vector Laboratories, REF: MP-7500) was used as the secondary antibody. To stain tissue sections, the ImmPact^®^ DAB Peroxidase (HRP) Substrate Kit was used per the manufacturer’s instructions (Vector Laboratories, REF: SK-4105). Hematoxylin was used as a counterstain. The antibodies used were anti-Cleaved Caspase 3 antibody (Asp175) (5A1E) (Cell Signaling, Danvers, MA, USA, REF: 9664, at a 1:1000 dilution) and Phospho-Histone H2A.X (Ser139) (20E3) (Cell Signaling, REF: 9718, 1:200). Tissue sections were imaged at 10× magnification after staining and quantified using ImageJ Fiji Version: 2.14.0/1.54f.

**Statistics.** Statistical analysis was performed using GraphPad Prism Version: 10.4.1 (532). One-way ANOVA and Sidak’s multiple-comparisons tests were performed for analysis of mutation frequencies and spectra, and unpaired *t*-tests were performed for immunohistochemistry, cleavage and DNA end-joining assays. Results with a *p* < 0.05 were considered significant. All results were reported as mean ± SD except for immunohistochemistry, which was reported as mean ± SEM. * indicates a *p*-value < 0.05, ** indicates a *p*-value < 0.01, *** indicates a *p*-value < 0.001, and **** indicates a *p*-value < 0.0001.

## 3. Results

**Z-DNA-induced mutations decrease or are unchanged with increasing age.** Male transgenic mice, aged to 2 months and 18 months, were used to investigate the effects of aging on Z-DNA-induced mutagenesis. These mice contain a chromosomally integrated recoverable mutation reporter with a Z-DNA-forming sequence (CG14) integrated into the *LacZ* gene. Specifically, 33 bp of the *LacZ* mutation reporter gene was replaced with a Z-DNA-forming sequence [27]. After reaching the target age, mice were euthanized and brain, liver, spleen, and testis tissues were harvested. These initial tissues of interest were chosen based on their replicative potential, since we have found both replication-dependent and replication-independent mechanisms of Z-DNA-induced genetic instability [26]. Specifically, the brain and the liver have lower proliferation rates, and the spleen and the testis have higher proliferation rates. The mutation reporter was then recovered from the mouse genome and ligated to form a shuttle vector that was transformed into DH10β bacteria indicator cells for blue–white screening to obtain mutation frequencies, where white colonies represent mutant colonies and blue colonies represent wild-type colonies.

**Figure 1:**
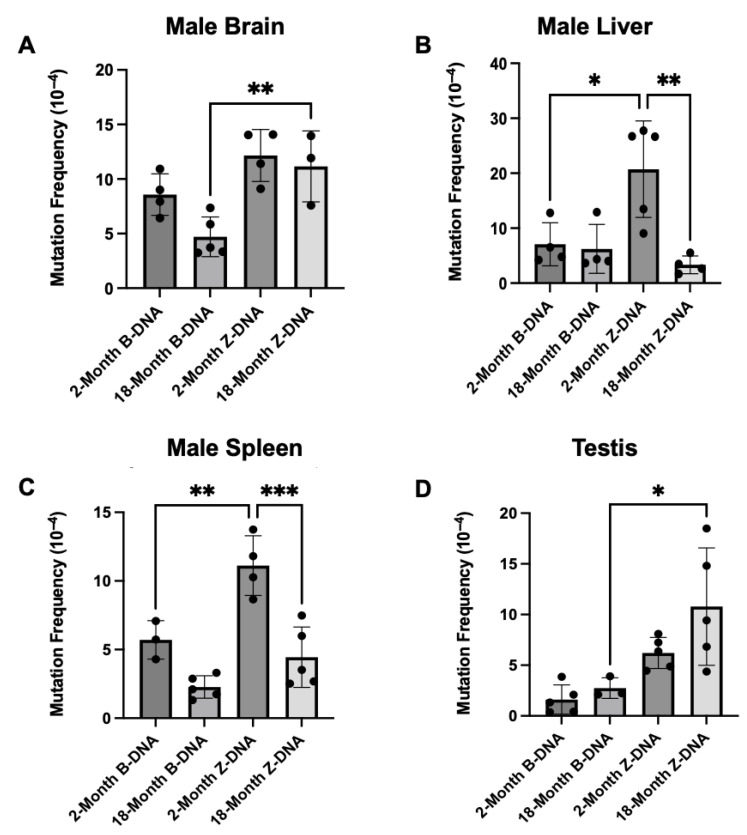
**Z-DNA-induced mutation frequencies in young and aged male mouse tissues.** (**A**) Z-DNA-induced mutation frequencies in male mouse brain tissues. (**B**) Z-DNA-induced mutation frequencies in male mouse liver tissues. (**C**) Z-DNA-induced mutation frequencies in male mouse spleen tissues. (**D**) Z-DNA-induced mutation frequencies in testis tissues. A total of 3–5 mice were used for each group, and a *p*-value < 0.05 was considered significant. * indicates a *p*-value < 0.05, ** indicates a *p*-value < 0.01, and *** indicates a *p*-value < 0.001.

The results revealed an induction of mutations in the Z-DNA mice over the B-DNA mice at 2 months of age, with significance being observed in the liver and the spleen (Figure 1). Specifically, a ~2.9-fold increase (*p* = 0.0137) in mutations was observed in the liver (Figure 1B) and a ~1.9-fold increase (*p* = 0.0056) in mutations was observed in the spleen over that of the control B-DNA mice (Figure 1C). This induction in mutations was lost once the mice reached 18 months of age, and instead, Z-DNA-induced mutation frequencies were reduced by ~6.2-fold (*p* = 0.0022) in the liver and ~2.5-fold in the spleen (*p* = 0.0003) compared to the mice at 2 months of age (Figure 1B,C). No significant changes in Z-DNA-induced mutation frequencies were observed in the male brain or testis with increasing age (Figure 1A,D).

To ascertain whether there were differences in de novo mutations between young and aged mice, primary skin fibroblasts were isolated from the mutation reporter mice at 2 months of age and mice at 18 months of age. Mutation reporter plasmids [pYZ-Con (B-DNA) or pYZ-CG14 (Z-DNA)] were transfected into the primary mouse skin fibroblasts to determine Z-DNA-induced mutation frequencies. We found that Z-DNA was more mutagenic than B-DNA in male primary skin fibroblasts from 2-month-old mice (~3.6-fold increase, *p* = 0.0308). Similar to our results from the mouse tissues, there was a decrease in Z-DNA-induced mutation frequencies in mouse skin fibroblasts taken from 18-month-old mice compared to those from 2-month-old mice (~1.8-fold decrease) (Supplementary Figure S1).

**Figure 2:**
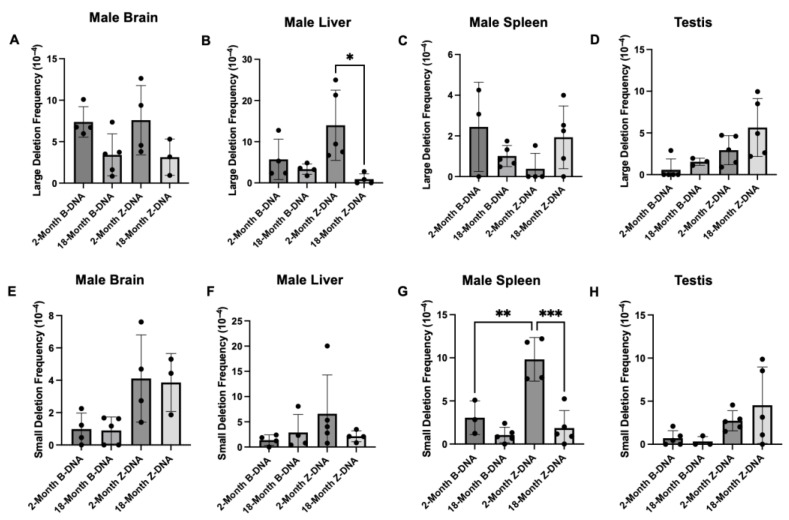
**Z-DNA-induced mutation spectra analyses in young and aged male mouse tissues.** Large deletion frequencies in male mouse brain (**A**), liver (**B**), spleen (**C**), and testis tissues (**D**). Small deletion frequencies in male mouse brain (**E**), liver (**F**), spleen (**G**), and testis tissues (**H**). A total of 3–5 mice were used in each group, and a *p*-value < 0.05 was considered significant. * indicates a *p*-value < 0.05, ** indicates a *p*-value < 0.01, and *** indicates a *p*-value < 0.001.

**Deletions were the predominant type of Z-DNA-induced mutation in mouse tissues.** DNA from white mutant bacteria colonies from the blue–white screening was isolated and sent for Sanger sequencing to ascertain the types of mutations induced by Z-DNA. Sequencing analysis revealed that large (>30 bp) and small (<30 bp) deletions were the two predominant types of Z-DNA-induced mutations in the mouse tissues tested (Figure 2 and Figure S2). While large and small deletions occurred in both the brain and the testis, no significant changes were observed with increasing age (Figure 2A,D,E,H). In the mouse liver tissue, there was a significant reduction in Z-DNA-induced large deletions with age (Figure 2B). In contrast, in the spleen tissue, there was a significant reduction in the frequency of Z-DNA-induced small deletions with increasing age (Figure 2G). In male primary mouse fibroblast cells, Z-DNA-induced large deletions occurred more frequently than small deletions. However, like the liver tissue, Z-DNA-induced large deletions in primary mouse fibroblasts decreased with increasing age (Supplementary Figure S1). Additional mutations such as base substitutions, insertions, and translocations were also observed in all tissues examined. These other types of mutations occurred at lower frequencies compared to large and small deletions, and no significant differences were observed with age (Supplementary Figure S2).

**Figure 3:**
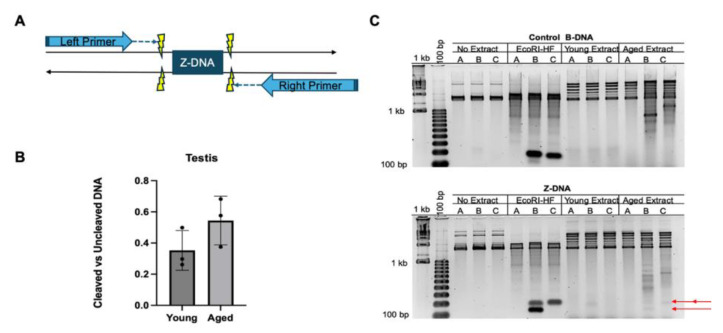
**Cleavage activity on Z-DNA in mouse testis tissue extract with increasing age.** (**A**) Schematic of the primer extension assay. Potential cleaved Z-DNA sites are marked by yellow lightning bolts. (**B**) Graphical representation of cleaved Z-DNA products with increasing age in mouse testis tissue extract, n = 3. (**C**) Gel image of cleaved products from the primer extension assay. Cleaved products are indicated by the red arrows. “A” represents no primers used, “B” represents right primer, and “C” represents left primer.

**Cleavage of Z-DNA is unchanged with increasing age.** A primer extension assay was performed to determine whether the cleavage of Z-DNA was altered with increasing age (Figure 3A). DNA substrates containing a Z-DNA-forming sequence (PNU-CG14) was incubated in whole testis tissue extract to allow proteins to interact with the Z-DNA structure. Subsequently, the plasmid DNA was purified and then amplified via primer extension using either a JMleft or a JMright primer to amplify cleaved Z-DNA products (Supplementary Table S1). Cleavage of Z-DNA was observed in testis extracts from both young and aged mice; however, there were no significant changes with increasing age (Figure 3B,C). This suggests that the recognition and cleavage of Z-DNA is unaltered with increasing age.

**Figure 4:**
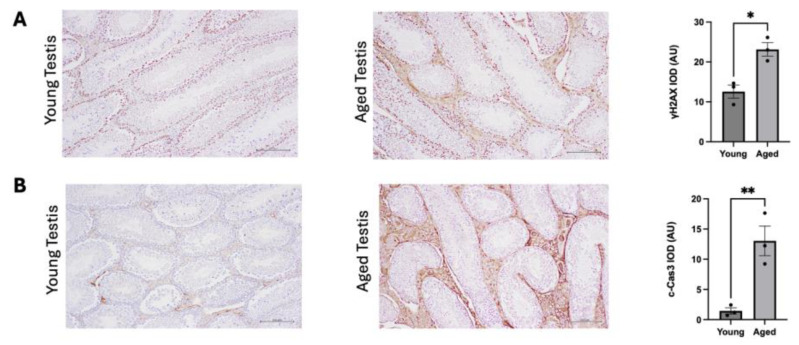
**Immunohistochemistry analysis of γH2AX and c-Cas3 in young and aged mouse testis tissues.** (**A**) Representative image of γH2AX foci formation in young and aged testis tissues (n = 3). The results are quantified in the graph on the right. (**B**) Representative image of c-Cas3 in young and aged testis tissues (n = 3). The results are quantified in the graph on the right. A *p*-value < 0.05 was considered significant. * indicates a *p*-value < 0.05 and ** indicates a *p*-value < 0.01.

**DNA end-joining proficiency decreases with increasing age.** Z-DNA has been shown to stimulate the formation of DSBs in yeast and mammalian cells [25,26]. These DSBs can be processed in a mutagenic fashion via microhomology-mediated end-joining mechanisms. This is supported by the presence of microhomologies at the Z-DNA-induced deletion breakpoints in mammalian cells, implicating microhomology-mediated end-joining in their processing [26]. To determine whether there is an increase in double-strand break formation with increasing age, testis tissue sections were stained for γH2AX foci formation. Significant increases in γH2AX foci were observed in the aged testis tissues compared to the young testis samples (~1.8-fold increase, *p* = 0.0110) (Figure 4A). Similarly, significant increases in cleaved caspase 3 (c-Cas3) were observed in aged testis samples (~9-fold increase, *p* = 0.100) (Figure 4B), indicating increases in cellular apoptosis. These two observations suggest a decrease in the efficiency in DSB repair pathways.

**Figure 5:**
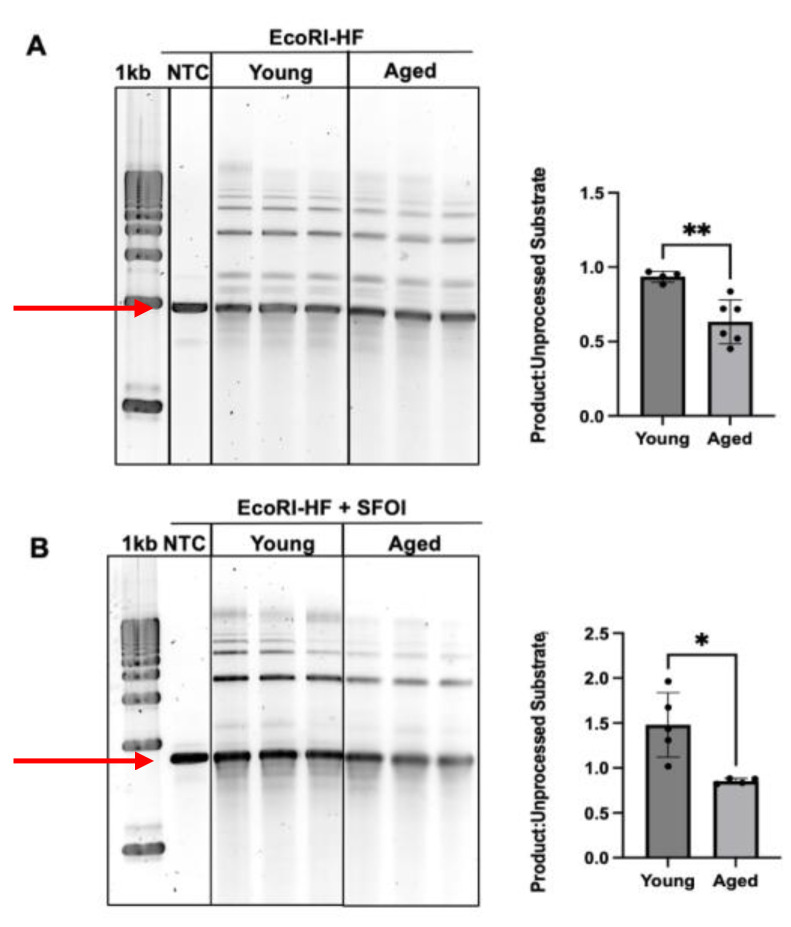
**DNA end-joining analysis in young and aged mouse testis tissue.** (**A**) End-joined DNA products formed in young and aged mouse testis extracts using DNA substrates with compatible overhanging ends created by EcoRI-HF. (**B**) End-joined DNA products in young and aged mouse testis extracts using DNA substrates with both an overhanging end and a blunt end. Unprocessed DNA substrates are represented by the red arrows. All DNA bands above the red arrows were considered end-joined products. A total of 4–6 mice were used for each group, and *p* < 0.05 was considered significant. * indicates a *p*-value < 0.05 and ** indicates a *p*-value < 0.01.

To determine whether DSB repair pathways are attenuated with increasing age, an end-joining assay was designed using DNA substrates with overhanging ends. This DNA substrate was constructed by digesting pNUCON with EcoRI-HF. Subsequently, the DNA substrate was incubated in testis tissue extract to allow proteins to end-join or “repair” them. Upon completion, significant decreases in end-joining activity were observed in the aged testis tissue extract compared to the young testis tissue extract (*p* = 0.0031) (Figure 5A). This experiment was then performed using another DNA substrate containing both an overhanging end and a blunt end. This DNA substrate was constructed by dual digesting pNUCON with both EcoRI-HF and SFOI, after which the large DNA fragment was gel purified (1786 bp). Similar to results from the first DNA substrate with two overhanging ends, significant decreases in end-joining activity were observed using this DNA substrate (*p* = 0.0173) (Figure 5B). Altogether, this decrease in end-joining activity may provide a possible explanation for the decrease in Z-DNA-induced mutation frequencies with increasing age (Figure 1).

## 4. Discussion

Non-B DNA structures such as Z-DNA have been implicated in a variety of age-related diseases, such as cancer [9,22,23]. This is largely due to the mutagenic potential of these DNA genomic elements [26,33,34]. Z-DNA has been shown to increase genetic instability in a host of biological organisms [20,25,27] and is associated with the development of translocation breakpoints in human cancer genomes [23]. Recently, a mechanism for the processing of Z-DNA structures was established whereby they are recognized by MSH2-MSH3 and are then cleaved by ERCC1-XPF to create DSBs [25]. Here, we explored the effects of age on the mutagenic processing of Z-DNA using male transgenic mutation reporter mice. These mice contain a *LacZ* gene with a Z-DNA-forming sequence, CG14, inserted into the mouse genome to measure Z-DNA-induced genetic mutations over time from 2 months to 18 months of age [27].

Brain, liver, spleen, and testis tissues from male mice were evaluated to determine the impact of aging on Z-DNA-induced mutagenesis. The results were unexpected in that Z-DNA-induced mutations either decreased or remained unchanged with increasing age (Figure 1). This variation in Z-DNA-induced mutations may be caused by the capacity for double-strand break repair in the tissues, as one study performed in rats observed variable nonhomologous end-joining (NHEJ) efficiencies in somatic tissues [35]. Few tissue-specific differences were observed in the Z-DNA-induced mutation spectra. The most common mutation that occurred were deletions, where small deletions were categorized at <30 bps and large deletions at >30 bps. Notably, a significant shift in the frequency of large deletions was observed in the male mouse liver tissue with age (Figure 2B). In contrast, a significant decrease in small deletions was observed in male mouse spleen tissue (Figure 2G). This may hint at tissue-specific DSB repair processes that are involved in the processing of Z-DNA. For example, Z-DNA-induced DSBs may be repaired by MMEJ in the liver, where large deletions were the predominant mutation type, and via NHEJ in the spleen, where small deletions were largely observed [36].

Cleavage of Z-DNA was investigated to determine whether the initial processing of Z-DNA was altered with increasing age [25], which could account for the decreases detected in Z-DNA-induced mutation frequencies. In the testis tissue, there were no changes in cleaved Z-DNA products between young and aged extracts (Figure 3B). This suggests that any potential changes in the processing of Z-DNA structures are occurring downstream of the initial recognition and cleavage events [25].

After the cleavage of Z-DNA by ERCC1-XPF, MMEJ mechanisms can process the Z-DNA-induced DSBs, which can result in deletions [25,26]. This is indicated by the occurrence of microhomologies in the Z-DNA-induced deletion breakpoint junctions [26,36]. The role of DSB repair processes was confirmed through the introduction of an NHEJ system into *E. coli*, where Z-DNA-induced mutations shifted from small deletions and insertions to large deletions [37]. To better understand the DNA repair mechanisms involved in the repair of Z-DNA-induced breaks following cleavage, the efficiency of DSB repair processes was explored through an end-joining assay where DNA substrates with varying ends were incubated into mouse testis tissue extracts. The results indicated that the end-joining efficiency decreased in aged tissues samples compared to in young tissue samples (Figure 5). This was further supported by the examination of γH2AX foci formation and c-Cas3 through immunohistochemistry, where there were significant increases in both γH2AX and c-Cas3 in aged testis compared to young testis tissues (Figure 4). Together, these results suggest an attenuation in DSB repair pathways in the aged mice, which may provide an explanation for the reduced Z-DNA-induced mutation frequencies in the 18-month-old mice compared to the 2-month-old mice. Indeed, other labs have found decreases in protein expression and kinetics in DSB repair pathways with increasing age in both mice and humans [38,39,40,41,42].

Here, we provide evidence that Z-DNA is mutagenic in vivo in mouse tissues. Surprisingly, we found that the Z-DNA-induced mutations either remained the same or decreased with age. We found that Z-DNA cleavage was not altered with age, end-joining capacity decreased with age, and DSBs and apoptosis increased with age. Thus, we propose that aging alters DSB repair processes and thereby impacts Z-DNA-induced mutations, as the recognition and cleavage of Z-DNA remains unchanged with increasing age. However, additional studies on the exact mechanism(s) involved in the mutagenic processing of Z-DNA with increasing age are warranted. By understanding the mechanism(s) by which age impacts Z-DNA-induced genetic instability and disease development, additional strategies on how to utilize Z-DNA structures can be developed for the treatment of age-related diseases such as cancer [43,44,45].

## Data Availability

The original contributions presented in this study are included in the article/Supplementary Materials. Further inquiries can be directed to the corresponding author.

## Supplementary Materials

The following supporting information can be downloaded at: https://www.mdpi.com/article/10.3390/genes16080942/s1, Table S1. List of oligonucleotides used in this study; Figure S1. Z-DNA-induced mutation frequencies and spectra in male primary mouse fibroblast cells as a function of age. (A) Z-DNA-induced mutation frequencies in male primary mouse fibroblast cells with increasing age. (B) Z-DNA-induced mutation spectra in male primary mouse fibroblast cells with increasing age; Figure S2. Z-DNA-induced mutation signature comparison in 2-month-old and 18-month-old male mouse tissues. (A) Comparison of the frequency of various Z-DNA-induced mutation signatures in 2-month-old male mouse tissues. (B) Comparison of the frequency of various Z-DNA-induced mutation signatures in 18-month-old male mouse tissues.
