# Supplementary material for: Effects of Aging on Z-DNA-Induced Genetic Instability In Vivo
Source: Genes (Basel). 2025 Aug 11;16(8):942. doi: 10.3390/genes16080942 (PMC12385340; doi:10.3390/genes16080942)
Supplement: Supplementary file 1 [file genes-16-00942-s001.zip › genes-3692229-supplementary.pdf]

Supplementary Table S1. List of oligonucleotides used in this study.

Namee	Sequence
Seqprim 189	5’ CAA AAA AGG GAA TAA GGG CG 3’
201 Primer	5’ CGT TTC TGG GTG AGC AAA A 3’
548 Primer	5’ GGT GAT GAC GGT GAA AAC CT 3’
JMleft	5’ GGA GAA AAT ACC GCA TCA GG 3’
JMright	5’ ATT AGG CAC CCC AGG CTT TA 3’

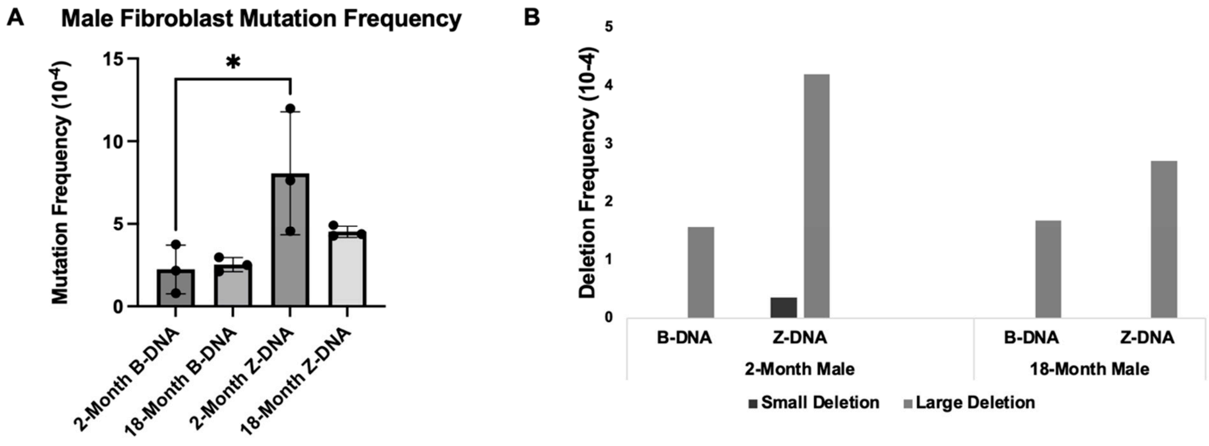

Supplementary Figure S1. Z-DNA-induced mutation frequencies and spectra in male primary mouse fibroblast cells as a function of age. (**A**) Z-DNA-induced mutation frequencies in male primary mouse fibroblast cells with increasing age. (**B**) Z-DNA-induced mutation spectra in male primary mouse fibroblast cells with increasing age.

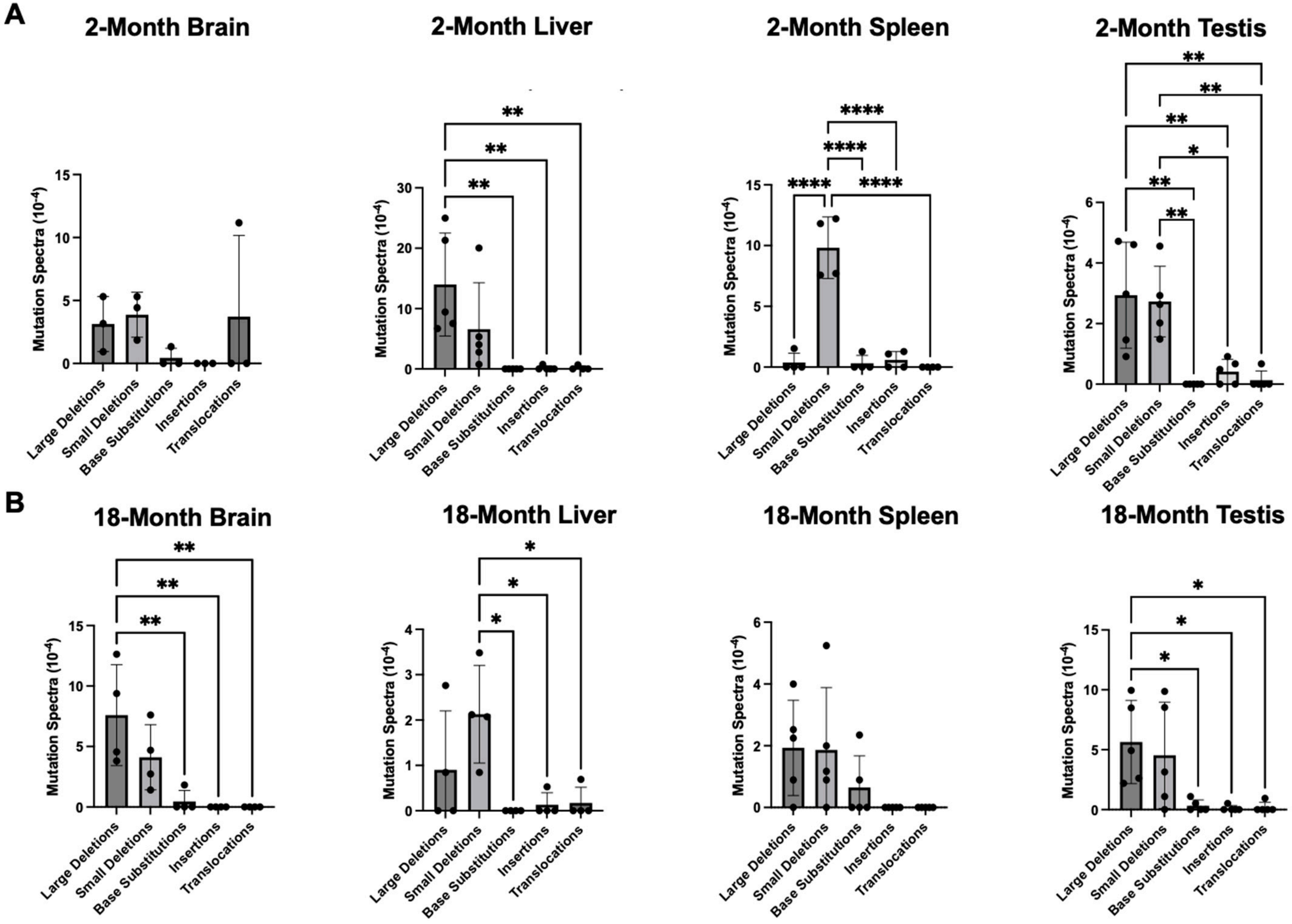

Supplementary Figure S2. Z-DNA-induced mutation signature comparison in 2-month-old and 18-month-old male mouse tissues. (**A**) Comparison of the frequency of various Z-DNA-induced mutation signatures in 2-month-old male mouse tissues. (**B)** Comparison of the frequency of various Z-DNA-induced mutation signatures in 18-month-old male mouse tissues.
